# CD40L Reverse Signaling Influences Dendrite Spine Morphology and Expression of PSD-95 and Rho Small GTPases

**DOI:** 10.3389/fcell.2020.00254

**Published:** 2020-04-28

**Authors:** Paulina Carriba, Sean Wyatt, Alun M. Davies

**Affiliations:** Neuron Development, Neurosciences Department, School of Biosciences, Cardiff University, Cardiff, United Kingdom

**Keywords:** CD40L reverse signaling, dendritic spines, PSD-95, Rho small GTPases, Rho, Cdc42

## Abstract

CD40-activated CD40L reverse signaling is a major physiological regulator of neural process growth from many kinds of developing neurons. Here we have investigated whether CD40L-reverse signaling also influences dendrite spine number and morphology in striatal medium spiny neurons (MSNs). Golgi preparations revealed no differences in the spine density, but because the dendrite arbors of MSNs were larger and branched in *Cd40*^–/–^ mice, the total number of spines was greater in *Cd40*^–/–^ mice. We also detected more mature spines compared with wild-type littermates. Western blot revealed that MSN cultures from *Cd40*^–/–^ mice had significantly less PSD-95 and there were changes in RhoA/B/C and Cdc42. Immunocytochemistry revealed that PSD-95 was clustered in spines in *Cd40*^–/–^ neurons compared with more diffuse labeling in *Cd40*^+/+^ neurons. Activation of CD40L-reverse signaling with CD40-Fc prevented the changes observed in *Cd40*^–/–^ cultures. Our findings suggest that CD40L-reverse signaling influences dendrite spine morphology and related protein expression and distribution.

## Introduction

Spines are small dynamic protrusions along the dendrites of many kinds of neurons that indicate the location of excitatory synapses. Changes in spine number and morphology occur throughout life and have a major bearing on the establishment and modification of the functional properties of neural circuits (Chidambaram et al., 2019). Dendritic spine changes are associated with a host of neurological disorders such schizophrenia (Glausier and Lewis, 2013), depression (Qiao et al., 2016), epilepsy (Wong and Guo, 2013), autism spectrum disorder (Joensuu et al., 2017), and neurodegenerative diseases (Deutch et al., 2007; Nithianantharajah and Hannan, 2013; Herms and Dorostkar, 2016; Pchitskaya et al., 2018). This emphasizes the need to better understand how dendritic spine number and morphology are regulated.

Medium spiny neurons (MSNs) comprise the great majority of neurons in the striatum. These GABAergic inhibitory neurons have exuberant dendritic arbors that are densely studded with spines. They receive extensive glutamatergic input from the cortex, hippocampus, amygdala, and thalamus and dopaminergic input from the ventral tegmental area and substantia nigra. They project in turn to the substantia nigra and globus pallidus (Gerfen, 1988; Yager et al., 2015). Because of their extensive and unique connections, MSNs play a central role in the control of voluntary movement, procedural memory, and motivated behavior (Natsubori et al., 2017). Degeneration and loss of MSNs is the main feature of Huntington’s disease and underlies the symptomology of this hereditary neurodegenerative disorder (Reiner et al., 1988). Changes in the size and morphology of MSN dendrite arbors and in the density and shape of their spines are associated with a range of neurological disorders, including Parkinson’s disease, dystonia, compulsivity, addiction, hyperactivity, and depression (Deutch et al., 2007; Crittenden and Graybiel, 2011; Kim et al., 2013; Shepherd, 2013; Villalba and Smith, 2013; Yager et al., 2015).

CD40-activated CD40L reverse signaling has recently been shown to be a major physiological regulator of MSN dendrite arbor size and complexity. MSN dendrite arbors are larger and more branched in *Cd40*^–/–^ mice *in vivo*, and *in vitro* experiments suggest that CD40-activated CD40L-mediated reverse signaling suppresses MSN dendrite growth and elaboration by a PKCγ-dependent mechanism (Carriba and Davies, 2017). Here we have investigated whether CD40L reverse signaling also influences MSN dendrite spine number and morphology. We observed alterations in the number and type of spines in *Cd40*^–/–^ mice. These morphological modifications were accompanied by changes in the expression of proteins that have been implicated in determining dendrite spine morphology, namely, PSD-95 and the Rho small GTPases RhoA/B/C and Cdc42. Phenotypic rescue experiments in culture suggested that these changes in *Cd40*^–/–^ mice were due to disruption of CD40L reverse signaling. Our findings suggest that CD40L reverse signaling regulates the expression and distribution of proteins implicated in governing spine morphology.

## Materials and Methods

### Mice

Mice were housed in a 12 h light-dark cycle with access to food and water *ad libitum*. Breeding was approved by the Cardiff University Ethical Review Board and was performed within the guidelines of the Home Office Animals (Scientific Procedures) Act, 1986. *Cd40* null mutant mice in a C57BL6/J background were purchased from The Jackson Laboratory (Maine, United States). These mice were backcrossed into a CD1 background. *Cd40*^±^ mice were crossed to generate *Cd40*^+/+^ and *Cd40*^–/–^ mice.

### Neuron Culture

Medium spiny neuron (MSN) cultures were prepared from E14 striatal primordia that were triturated to produce a single cell suspension following trypsin digestion (Worthington, Lakewood, United States) and DNase I treatment (Roche Applied Science, East Sussex, United Kingdom). Neurons were plated in plastic dishes pre-coated with poly-L-lysine (Sigma-Aldrich, Dorset, United Kingdom) at a density of 15,000 cells/cm^2^ for immunocytochemistry labeling and a density of 20,000 cells/cm^2^ for immunoblotting experiments. Neurons were cultured with Neurobasal A (Invitrogen, Paisley, United Kingdom) supplemented with 2% NeuroCult SM1 neuronal supplement (StemCell, Cambridge, United Kingdom), 1% Foetal Calf Serum (FCS) (Sigma-Aldrich, Dorset, United Kingdom), 100 units/ml penicillin, and 100 μg/ml streptomycin (Gibco BRL, Crewe, United Kingdom). To avoid extensive astrocyte proliferation, the medium was changed to medium without FCS after 5 days *in vitro* and then partially replaced every 4–5 days.

When indicated in the text, the cultures were treated the day after seeding with Fc protein (1 μg/ml) or CD40-Fc (1 μg/ml) (ALX-203-004-C050 and ALX-522-016-C050, respectively, from Enzo Life Sciences). Both proteins were reconstituted with sterile H_2_O as indicated in the datasheet.

### Golgi Preparations and Analysis of Neurite Spine Morphology

Modified Golgi-Cox impregnation was performed on 100 μm parasagittal sections of the striatum of P10 mouse brains of *Cd40*^+/+^ and *Cd40*^–/–^ littermates using the FD Rapid GolgiStain kit (FD NeuroTechnologies, Ellicott City, MD, United States).

For the analysis of neurite spine morphology, preparations were visualized using a Zeiss Axio Vert.A1 microscope with 100X oil immersion objective. Fifty micrometer dendrite fragments, separated from the soma between 10 and 20 μm, with the spines in a similar flat level were randomly selected for the quantifications. Graphs show the mean and standard errors of the mean of 118 separate 50 μm-fragments obtained from preparations of at least three mice of each genotype. For the analysis of neurite spine morphology in cultured neurons, the procedure was similar to the Golgi preparations. Graphs show the mean and standard errors of the mean of 50 μm-fragments obtained from at least three independent cultures (separate 50 μm-fragments counted for *Cd40*^+/+^ : 48; *Cd40*^–/–^: 63; *Cd40*^–/–^ treated with Fc: 41 and *Cd40*^–/–^ treated with CD40-Fc: 68). Spines were counted and classified based on established morphology (branched, mushroom, stubby, thin, and filopodia) (Dailey and Smith, 1996; Peters and Kaiserman-Abramof, 1970; Lendvai et al., 2000; Portera-Cailliau et al., 2003; Risher et al., 2014).

### Immunoblotting

Dissected striatal tissue from *Cd40*^+/+^ and *Cd40*^–/–^ was placed in triton lysis buffer supplemented with protease and phosphatase inhibitor cocktail mix (Protease/Phosphatase inhibitor cocktail, 5872, Cell Signaling). Tissue was disaggregated using a pellet pestle until complete homogenization. Cultured neurons were scraped out of the plates in ice-cold PBS, collected by centrifugation, and resuspended in ice-cold supplemented triton lysis buffer. After protein quantification for equal loadings, proteins were separated on 10% SDS-PAGE gels and were transferred to PVDF membranes (Immobilon-P, Millipore, United Kingdom). The blots were probed with anti-PSD-95 (1:1,000; rabbit 2507, Cell Signaling), anti-Synaptophysin (1:1,000; rabbit 5467, Cell Signaling), anti-Cdc42 (1:1,000; rabbit 2466, Cell Signaling) and anti-Rac1/2/3 (1:1,000; rabbit 2465, Cell Signaling), anti-RhoA/B/C (1:2,000; rabbit ab188103, AbCam), anti-GAPDH (1:90,000; mouse G8795, Sigma) and anti-βIII tubulin (1:90,000; mouse MAB1195, R&D). Binding of the primary antibodies was visualized with HRP-conjugated donkey anti-rabbit or anti-mouse secondary antibodies (1:5,000; rabbit W4011, mouse W4021, Promega, Southampton, United Kingdom) and EZ-ECL kit Enhanced Chemiluminescence Detection Kit (Biological Industries, Geneflow Limited, Staffordshire, United Kingdom). Densitometry was carried out using ImageJ software (NIH). The intensities of the proteins analyzed were normalized to the βIII tubulin band.

### Immunohistochemistry and Immunocytochemistry

For immunohistochemistry, P10 brains were fixed in fresh 4% paraformaldehyde in 0.12 M phosphate buffer, pH 7.2 for 24 h at 4°C. After washing in PBS, the tissue was cryoprotected in 30% sucrose before being frozen. Tissue was frozen in isopentene cooled with dry ice and was serially sectioned in the parasagittal plane at 30 μm. After washing with PBS, the sections were permeabilizated with 0.1% Triton X-100 for 1 h and then blocked with 1% BSA and 0.1% Triton X-100 (Sigma-Aldrich, Dorset, United Kingdom) in PBS for 2 h at room temperature.

For immunocytochemistry, cultures were fixed as above for 15 min at room temperature and were washed extensively with PBS before permeabilization and blocking of non-specific binding for 1 h at room temperature as indicated for immunohistochemistry.

For both procedures, primary antibodies were prepared in PBS with 0.5% BSA and 0.1% Triton X-100 and incubated overnight at 4°C. After washing in PBS, the immunohistochemistry sections or the neuron cultures were incubated for 2 h with secondary fluorescent Alexa antibodies prepared as before, washed extensively with PBS, and mounted on slices with Fluoromount-G (Cambridge Bioscience, United Kingdom). Images were obtained with Zeiss LSM710 confocal microscope.

Primary antibodies used were: anti-PSD-95 (1:100; rabbit 2507), anti-Cdc42 (1:100; rabbit 2466) and anti-Rac1/2/3 (1:100; rabbit 2465) from Cell Signaling, anti-DARPP-32 (1:100; rat MAB4230, R&D Systems), and anti-RhoA/B/C (1:500; rabbit ab188103, AbCam). Secondary fluorescent Alexa antibodies used were donkey anti-rabbit Alexa 594 and goat anti-rat Alexa 488 (1:500, Thermofisher, United Kingdom; A-21207 and A-11006). Negative controls without primary antibody were also set up (not shown).

## Results and Discussion

### Phenotypic Changes in the Dendrite Spines of MSNs of *Cd40*^–/–^ Mice

**FIGURE 1:**
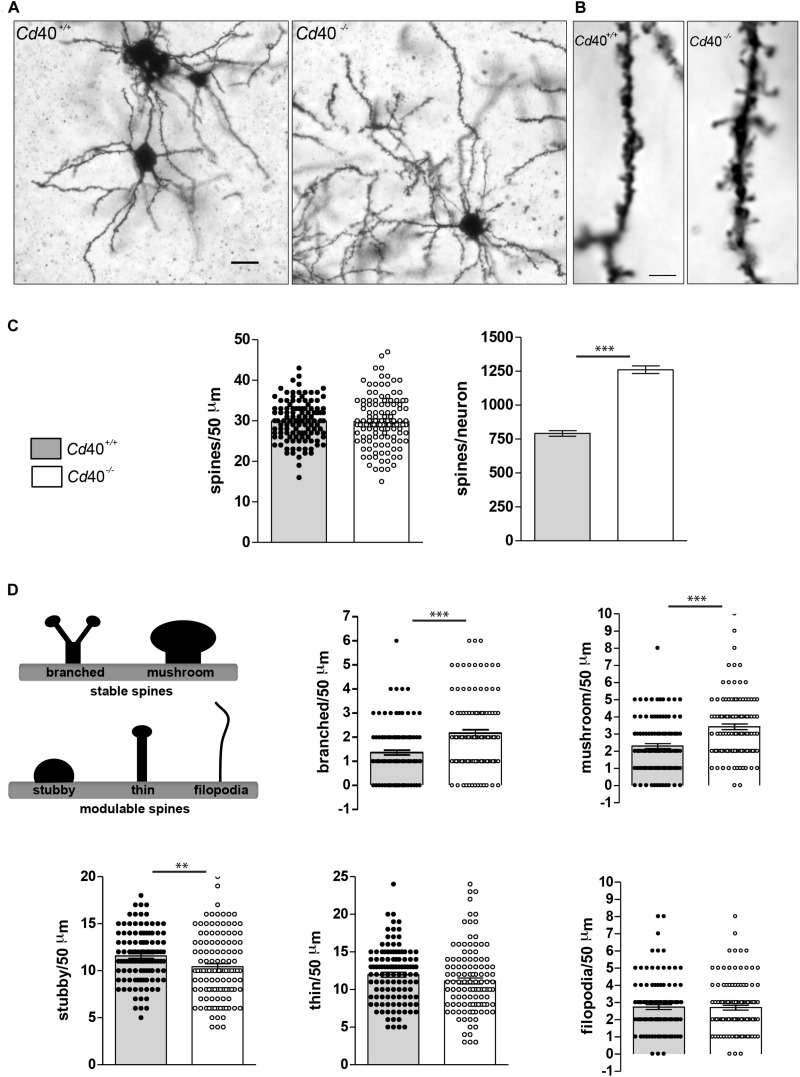
Phenotypic changes in striatal MSN spines in p10 *Cd40*^+/+^ and *Cd40*^–/–^ mice. **(A)** Illustrative photomicrographs of MSNs from P10 *Cd40*^+/+^ and *Cd40*^–/–^ mice stained by modified Golgi-Cox impregnation. Scale bar, 20 μm. **(B)** Representative high-resolution (100X oil objective) photomicrographs of spines from P10 *Cd40*^+/+^ and *Cd40*^–/–^ mice in 50 μm dendrite fragments. Scale bar, 5 μm. **(C)** Quantification of total number of spines per 50 μm dendrite fragment; and estimation of the total number of spines per neuron. **(D)** Classification of type of spines: mature stable spines (branched and mushroom) and immature modulable spines (stubby, thin and filopodia). Quantification of the types of spines per 50 μm dendrite fragment. Branched and mushrooms (upper charts) and stubby, thin and filopodia (bottom charts). The means ± s.e.m from 118 separately 50 μm-fragments obtained from preparations of at least three mice of each genotype are shown in the graphs. The dots represent the total number of spines counted. *T*-test ^∗∗∗^*p* < 0.0001 and ^∗∗^*p* < 0.001.

To determine whether CD40 affects spine number and morphology in developing MSNs, we analyzed Golgi preparations of the striatum of *Cd40*^–/–^ and *Cd40*^+/+^ littermates. These studies were carried on postnatal day 10 (P10) animals, an age at which we have shown that *Cd40* deletion has a marked and striking effect on MSN dendrite size (Carriba and Davies, 2017). Low power images confirmed that the dendrite arbors of MSNs in *Cd40*^–/–^ mice were very much larger and more exuberant than those of *Cd40*^+/+^ mice (Figure 1A). Spine number per unit dendrite length was quantified in high power images (Figure 1B) of 50 μm dendrite lengths chosen at random, beginning between 10 and 20 μm from the soma of numerous MSNs. These counts revealed that there was no significant difference in spine density between MSNs of *Cd40*^–/–^ and *Cd40*^+/+^ mice (Figure 1C, left bar chart). However, because MSN dendrites were longer and more branched in *Cd40*^–/–^ mice (total dendrite length per neuron: *Cd40*^+/+^ = 1321.74 ± 49.44 μm and *Cd40*^–/–^ = 2112.08 ± 68.45 μm) there were ∼60% more spines in the MSNs of *Cd40*^–/–^ mice than *Cd40*^+/+^ mice. This difference in the total number of spines per MSN (791.49 ± 20.71 in *Cd40*^+/+^ mice vs. 1261.16 ± 27.61 in and *Cd40*^–/–^ mice) was highly significant (Figure 1C, right chart).

In addition to assessing spine density and number, we also classified each spine into one of five types based on standard, accepted criteria (Dailey and Smith, 1996; Peters and Kaiserman-Abramof, 1970; Lendvai et al., 2000; Portera-Cailliau et al., 2003; Risher et al., 2014). These standard types (branched, mushroom, stubby, thin, and filopodia) are illustrated in the scheme shown in Figure 1D. Blind analysis revealed that MSNs of *Cd40*^–/–^ mice had significantly more mushroom and branched type spines and significantly fewer stubby types than those of *Cd40*^+/+^ mice (Figure 1D). There were no significant differences in the proportion of filopodia and thin spines between MSNs of *Cd40*^–/–^ and *Cd40*^+/+^ mice (Figure 1D). These finding suggest that CD40 has a striking effect on the morphology of MSNs dendrite spines. Because branched and mushroom spines are generally considered to be the more mature type of spine, it seems that absence of CD40 leads to a greater proportion of mature spines.

### Changes in the Expression and Distribution of PSD-95 in *Cd40*^–/–^ Mice

To understand how CD40 might influence dendrite spine morphology, we compared the expression of proteins that have been implicated in regulating spine morphology. One such protein is PSD-95 (postsynaptic density protein 95), also known as synapse-associated protein 90 (SAP-90) or disks large homolog 4 (DLG4). This is expressed in the postsynaptic density (PSD) and has been shown to play a key role in spine maturation and stabilization. Immunocytochemical studies have previously reported that dynamic and plastic spines (learning spines) express low levels of PSD-95, whereas PSD-95 accumulates in stable spines that have formed strong synaptic connections (memory spines) (El-Husseini et al., 2000; Ehrlich et al., 2007; Berry and Nedivi, 2017).

**FIGURE 2:**
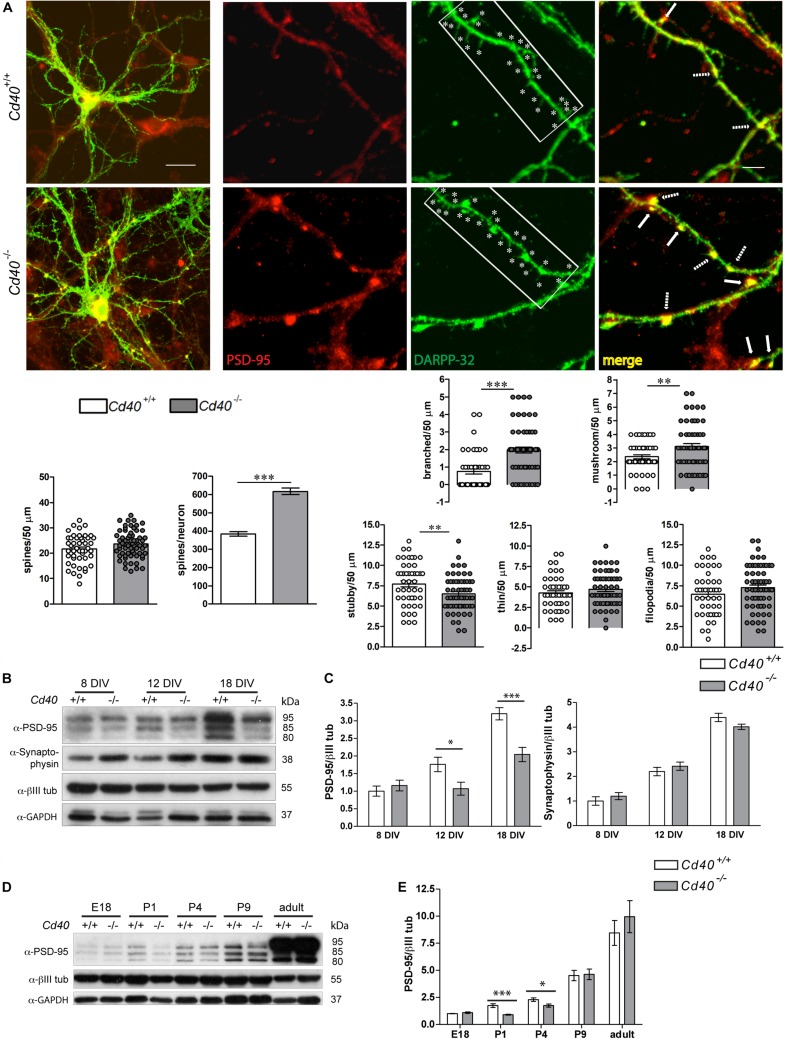
Expression of PSD-95 in striatal *Cd40*^+/+^ and *Cd40*^–/–^ mice tissue and neurons in culture. **(A)** Immunocytochemical localization of PSD-95 (red), DARPP-32 (green) and merge (yellow) in *Cd40*^+/+^ (upper panels) and *Cd40*^–/–^ (lower panels) striatal neurons from E14 embryos cultured 18 days. In the DARPP-32 images, * indicates spines in 40 μm fragments. In the merge images, the arrows indicate the co-localization of PSD-95 and DARPP-32 in spines and the dashed arrows indicate the co-localization of these proteins in the base of spines. Scale bars 20 and 5 μm. Quantification of total number of spines per 50 μm dendrite fragment; and estimation of the total number of spines per neuron. Quantification of the types of spines per 50 μm dendrite fragment. Branched and mushrooms (upper charts) and stubby, thin and filopodia (bottom charts). The means ± s.e.m from 50 μm-fragments obtained from at least three independent cultures of *Cd40*^+/+^ (48, white bars) and *Cd40*^–/–^ (63, gray bars). The dots represent the total number of spines counted. *T*-test ***p < 0.001 and **p < 0.01. **(B)** Illustrative Western blots of the expression of PSD-95 and synaptophysin in cultured neurons from E14 embryo striatal tissue of *Cd40*^+/+^ and *Cd40*^–/–^ mice cultured the indicated days. **(C)** Quantification of the expression of these proteins from three Western blots from independent cultures. **(D)** Representative Western blot of the expression of PSD-95 in striatal tissue from *Cd40*^+/+^ and *Cd40*^–/–^ at the indicated ages. **(E)** Quantification from three independent Western blots of the expression of PSD-95 in samples from striatal tissue. **(B,D)** Expression of βIII tubulin and GAPDH were used as loading controls. **(C,E)** Bar graphs show the relative densitometry quantifications for each protein normalized to βIII tubulin (mean ± s.e.m). One-way ANOVA with multiple Newman-Keuls statistical comparison. ***p < 0.001, **p < 0.01, and *p < 0.05.

To explore the potential influence of CD40 on the localization of PSD-95 in striatal MSNs, we used immunocytochemistry to study the distribution of PSD-95 in DARPP-32-positive neurons in dissociated cultures established from the striata of *Cd40*^+/+^ and *Cd40*^–/–^ mice. Although more than 95% of the neurons in the striatum are MSN’s, cultures established from striata may contain some contamination by other neighboring neuronal types. PSD-95 is potentially expressed by all type of neurons, while DARPP-32 is a marker of this type of GABAergic neuron (Ouimet et al., 1998). Low-power images (Figure 2A, left) showed that these cultures replicated our previous report that the dendritic arbors of DARPP-32-positive neurons of *Cd40*^–/–^ mice are more exuberant than those of *Cd40*^+/+^ mice (Carriba and Davies, 2017). High-power images (Figure 2A, right) revealed that DARPP-32, which is preferentially clustered into the postsynaptic densities of the head and neck of spines of mature striatal neurons (Blom et al., 2013), occurs at intervals along the dendrites in spine-like structures. Moreover, PSD-95 staining was more intense in these larger spines than in the small spines observed in cultures established from *Cd40*^+/+^ mice. The co-localization of PSD-95 staining with DARPP-32 staining in the larger spine-like structures in cultures established from *Cd40*^–/–^ mice is particularly clear in the merged images, where co-localization is indicated by yellow staining. These observations suggest that there are more mature spines in *Cd40*^–/–^ cultures. Quantification of the number of spines in cultured neurons corroborated this observation. Left bar charts (Figure 2A, bottom) show the quantification of the number of spines per 50 μm-fragment unit and per neuron. As in spine quantifications of Golgi preparations, there was no significant difference in spine density between cultures established from *Cd40*^+/+^ and *Cd40*^–/–^. But because the neurons from *Cd40*^–/–^ have longer, more branched dendrites (1308.61 ± 53.96 μm total dendrite length per neuron) compared with neurons from *Cd40*^+^^/^^+^ mice (883.71 ± 36.48 μm total dendrite length per neuron), the total number of spines per neuron was significantly increased in neurons from *Cd40*^–/–^ mice (617.67 ± 18.11 spines per μm) compared with neurons from *Cd40*^+/+^ mice (383.53 ± 12.26 spines per μm). This equates to an ∼60% increase in the total number of spines per neuron in MSN cultures established from *Cd40*^–/–^ mice. Quantification of the type of spines in cultured neurons revealed similar results as *in vivo*, with significantly more branched and mushroom type spines (Figure 2A, top right bar charts), and less stubby spines, with no significant differences in thin and filopodia spines (Figure 2A, bottom right bar charts). These results are consistent with our *in vivo* observations that there are more morphologically mature spines in the striatum of *Cd40*^–/–^ mice.

In addition to differences in the distribution of PSD-95 in cultures established from *Cd40*^–/–^ and *Cd40*^+/+^ mice, we used Western blotting to ascertain whether there were differences in the total levels of PSD-95 in these cultures. The mouse expresses three isoforms of PSD-95 (PSD-95α, PSD-95β, and PSD-95γ) that are generated by alternative splicing and post-transcriptional modifications. These three isoforms are trafficked and clustered in different ways into PSDs (Chetkovich et al., 2002), suggesting that they could have different functions that are independently regulated. These isoforms can be seen in the representative Western blot shown in Figure 2B. These studies revealed that there were significantly lower levels of PSD-95 in *Cd40*^–/–^ cultures compared with *Cd40*^+/+^ cultures after 12 days *in vitro*, especially in the 80 and 85 kDa isoforms, suggesting that these isoforms may be more sensitive to the lack of CD40. This difference became highly significant after 18 days *in vitro*. To determine whether this difference in PSD-95 expression between *Cd40*^–/–^ and *Cd40*^+/+^ cultures was non-specific, we also assessed the levels in these cultures of another synaptic protein, synaptophysin. Western blotting revealed no significant difference in the levels of synaptophysin between *Cd40*^–/–^ and *Cd40*^+/+^ cultures after 8, 12, and 18 days *in vitro* (Figure 2C).

To ascertain whether the decrease in the total PSD-95 level observed in *Cd40*^–/–^ cultures was a consequence of an *in vitro* artifact, we used Western blotting to assess the relative levels of PSD-95 in striata dissected from *Cd40*^–/–^ and *Cd40*^+/+^ mice at several stages (E18, P1, P4, P9 and adult). These studies reveal a gradual increase in the total level of PSD-95 throughout development, but with significantly lower levels in the striata of *Cd40*^–/–^ mice relative to the striata of *Cd40*^+/+^ mice at P1 and P4. These results suggest that the absence of CD40 results in reduced expression of PSD-95 during a window of development and are consistent with our *in vitro* analysis (Figures 2D,E).

While there have been no studies that directly relate the overall quantity of PSD-95 to synaptic maturity, a decrease in the overall tissue level of PSD-95 would be predicted as something that accelerates synapse maturation, suggesting that turnover of PSD-95 is reduced as spines mature. Consistent with this, the mouse model of Huntington’s disease zQ125 has reduced levels of PSD-95 (Peng et al., 2016) and accelerated spine maturation (Mc McKinstry et al., 2014) in the striatal tissue compared to age-matched wild type animals. Detailed analysis with timeSTAMP (Time – Specific Tag for the Age Measurement of Proteins), which allows new copies of a genetically labeled protein to be labeled and followed, has shown that newly synthetized PSD-95 has synapse-autonomous functions and is not just synthetized to replenish the consumed proteins during synaptic plasticity (Butko et al., 2012). New PSD-95 is diffusely located in the cytosol and in dendritic protrusions and is less abundant in mature spines, while old copies of PSD-95 are preferentially observed clustered in mature spines with a slow turnover (Butko et al., 2012). Because the tissue level of PSD-95 is not correlated with its accumulation in clusters in stable dendritic spines (Okabe et al., 2001; Steiner et al., 2008; Sturgill et al., 2009; Fortin et al., 2014) and because PSD-95 has a very low turnover and is highly stable when it accumulates in mature spines (Ehlers, 2003; Gray et al., 2006; Yoshii and Constantine-Paton, 2007; Steiner et al., 2008; Sturgill et al., 2009; Fortin et al., 2014) it is likely that a decrease in the total level of PSD-95 would be associated with spine maturation. Moreover, the appropriate quantity of PSD-95 is required for the regulation of synaptic strength and for functional activity dependent synapse stabilization (Ehrlich et al., 2007). Future electrophysiological experiments will be needed to determine whether CD40-deficient dendritic spines are really mature functional spines.

### Differences in the Expression of Rho Small GTPases Between *Cd40*^+/+^ and *Cd40*^–/–^

The formation and ongoing plasticity of dendritic spines depends on the function of the actin cytoskeleton. Small GTPases are key regulators of the actin cytoskeleton that are switched on or off by exchange of GTP and GDP. This superfamily of proteins includes Ras, Rho, Rab, Arf/Sar1, and Ran. The Rho small GTPase family comprises 20 members distributed into 8 subfamilies (Rho, Rnd, RhoD/F, RhoH, Rac, Cdc42, RhoU/V, and RhoBTB). They play important roles in regulating actin cytoskeleton dynamics, including axon and dendrite growth and branching and spine morphology (Etienne-Manneville and Hall, 2002; Boureux et al., 2007). Rho, Rac, and Cdc42 are the most studied members from this family (Luo, 2000; Bonhoeffer and Yuste, 2002) and play a major role in regulating several aspects of spine dynamics. Rac has been implicated in spine proliferation and stabilization (Nakayama et al., 2000; Meng et al., 2002; Penzes et al., 2003), Rho in spine destabilization, retraction, and loss (Tashiro et al., 2000; Murakoshi et al., 2011), and Cdc42 in the control of spine length (Scott et al., 2003; Murakoshi et al., 2011).

The function of Rho small GTPases is dependent on changes in their localization and on many regulators and effectors. Inactive Rho small GTPases are located in the cytosol and translocate to the plasma membrane when activated. However, we did not observe any obvious difference in the localization of Rho small GTPases between striatal sections of P10 *Cd40*^+/+^ and *Cd40*^–/–^ mice (Supplementary Figure S1A). Likewise, 18 days *Cd40*^–/–^ cultures treated with either CD40-Fc or Fc showed no evident differences in the translocation of these proteins to the plasma membrane (Supplementary Figure S1B). Despite these negative results, we cannot exclude the possibility that CD40 affects Rho small GTPases activation.

**FIGURE 3:**
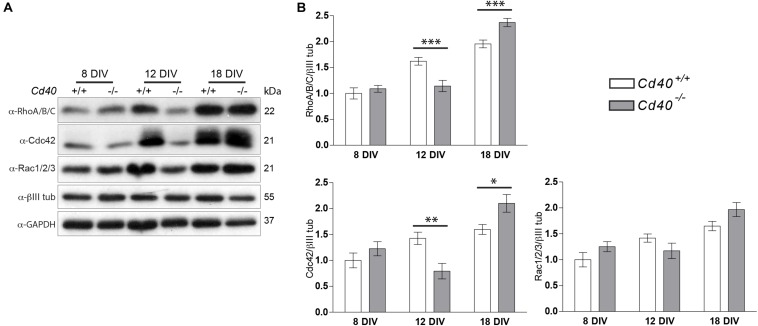
Expression of RhoA/B/C, Cdc42, and Rac1/2/3 in MSNs cultures from E14 embryo striatal tissue of *Cd40*^+/+^ and *Cd40*^–/–^ mice. **(A)** Representative Western blots of the expression of RhoA/B/C, Cdc42, and Rac1/2/3 in cultured neurons the indicated days. **(B)** Quantification of the relative expression of these proteins normalized to βIII tubulin from Western blots from at least three independent cultures. Graphs show mean ± s.e.m. One-way ANOVA with multiple Newman-Keuls statistical comparison. ***p < 0.001, **p < 0.01, and *p < 0.05.

We did, however, detect differences in the levels of these proteins in MSN cultures established from *Cd40*^+/+^ or *Cd40*^–/–^ embryos. Quantification of numerous independent Western blots revealed significantly lower levels of RhoA/B/C and Cdc42 in *Cd40*^–/–^ cultures compared to *Cd40*^+/+^ cultures after 12 days *in vitro* and significantly higher levels of these proteins in *Cd40*^–/–^ cultures after 18 days *in vitro* compared with the levels in *Cd40*^+/+^ cultures. There were no differences in the levels of these proteins between *Cd40*^+/+^ and *Cd40*^–/–^ cultures after 8 days *in vitro* and no significant differences in the levels of Rac1/2/3 in these cultures after 8, 12, or 18 days *in vitro* (Figures 3A,B). Rho small GTPases regulate several processes associated with actin dynamics, including organelle development and cytoskeletal dynamics. The elaboration of neural processes as neurons mature is associated with increased actin dynamics and an increase in the levels of Rho small GTPases. In *Cd40*^+/+^ cultures, the expression of all three Rho small GTPases analyzed increased. However, RhoA/B/C and Cdc42 expression levels did not increase in *Cd40*^–/–^ cultures between 8 and 12 days *in vitro*, but did increased abruptly between 12 and 18 DIV. This might reflect altered neuronal growth when CD40 is absent.

To investigate whether changes in the levels of RhoA/B/C and Cd42 proteins were due to transcriptional changes, we performed quantitative PCR (qPCR) to analyze whether there were changes in the levels of the respective mRNAs. In our experimental conditions, we did not detect any significant changes in the mRNA levels for these proteins (Supplementary Figure S2). This suggests that the changes in protein levels observed in *Cd40*^–/–^ culture were not due to transcriptional changes in the encoding genes, rather they were due to changes in the translation of mRNAs into proteins.

### Changes in PSD-95, RhoA/B/C, and Cdc42 Expression in *Cd40*^–/–^ Cultures Is Due to Absence of CD40L Reverse Signaling

Interaction between CD40L and CD40 can initiate bidirectional signaling: CD40L-activated CD40-mediated forward signaling and CD40-activated CD40L-mediated reverse signaling (Sun and Fink, 2007). In CD40 null animals, both signaling modalities are disrupted. Because CD40L reverse signaling is a major regulator of dendrite growth and elaboration in the CNS (Carriba and Davies, 2017), we speculated that this mechanism may also influence the expression of proteins that regulate spine growth and spine morphology. To test this, we cultured MSNs from E14 of *Cd40*^–/–^ embryo with either a CD40-Fc chimera (in which the extracellular domain of CD40 is linked to the Fc part of human IgG1) or an Fc control protein. This approach clearly defines the direction of the signaling: forward or reverse. If the regulation of spine growth and spine morphology is mediated by reverse signaling, the addition of the recombinant receptor to cultured cells from *Cd40*^–/–^ embryo will restore the wild type phenotype. However, if this is mediated by forward signaling, the addition of CD40-Fc to cells from *Cd40*^–/–^ animals will not have any effect.

**FIGURE 4:**
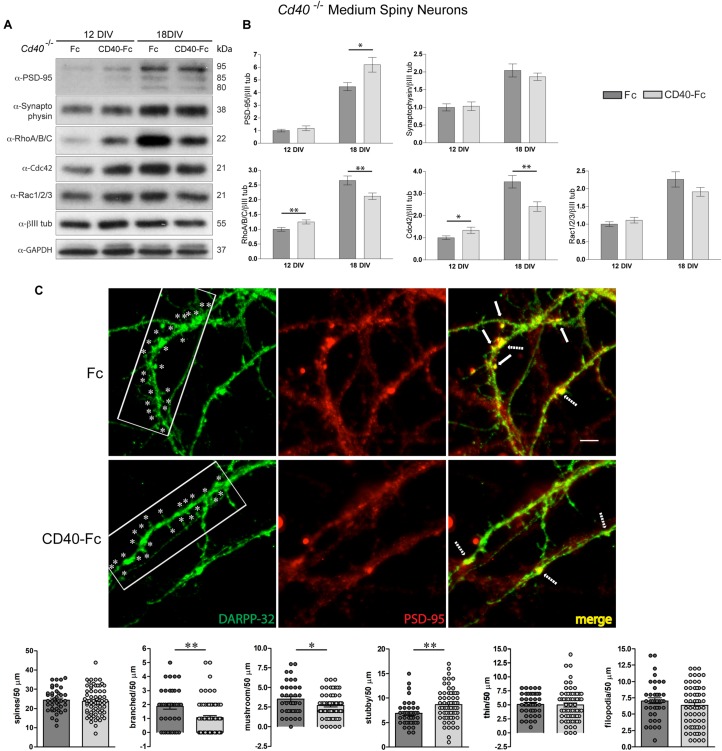
CD40L reverse signaling restores the expression of PSD-95, RhoA/B/C, and Cdc42 and resembles the spine morphology in *Cd40*^–/–^ cultured neurons. **(A)** Illustrative Western blots of the expression of PSD-95, synaptophysin, RhoA/B/C, Cdc42, and Rac1/2/3, using anti-βIII tubulin and anti-GAPDH as loading controls in *Cd40*^–/–^ cultured neurons treated with Fc or CD40-Fc 24 h after seeding and cultured during the indicated days. **(B)** Quantification of the relative expression of these proteins normalized to βIII tubulin from Western blots from at least three independent cultures. Graphs show mean ± s.e.m. One-way ANOVA with multiple Newman-Keuls statistical comparison. ^∗∗^*p* < 0.01 and ^∗^*p* < 0.05. **(C)** Immunocytochemical localization of DARPP-32 (green), PSD-95 (red), and merge (yellow) in 18 days cultures of *Cd40*^–/–^ treated 24 h after seeding with Fc or CD40-Fc. In the DARPP-32 images, ^∗^ indicates spines in 40 μm fragments. In the merge images, the arrows indicate the co-localization of PSD-95 and DARPP-32 in spines and the dashed arrows indicate co-localization of these proteins in the base of spines. Scale bar 5 μm. Quantification of total number of spines and of the type of spines (branched, mushroom, stubby, thin, and filopodia) per 50 μm dendrite fragment. The means ± s.e.m from 50 μm fragments obtained from at least three independent cultures of *Cd40*^–/–^ treated with Fc (41, dark gray bars) and CD40-Fc (68, pale gray bars). The dots represent the total number of spines counted. *T*-test ^∗∗^*p* < 0.01 and ^∗^*p* < 0.05.

The aim of these experiments was to ascertain whether the CD40-Fc chimera, which has been shown to activate CD40L-mediated reverse signaling (McWilliams et al., 2015; Carriba and Davies, 2017), could reverse the changes in protein expression and spine morphology observed in *Cd40*^–/–^ cultures. As shown in the representative Western blots (Figure 4A) and densitometry (Figure 4B), the addition of CD40-Fc chimera, but not the Fc protein, reversed the changes in the levels of RhoA/B/C and Cdc42 observed in *Cd40*^–/–^ cultures after 12 and 18 days *in vitro* and partially reversed the changes in the levels PSD-95 observed in *Cd40*^–/–^ cultures after 18 days *in vitro*. The levels of synaptophysin were unaltered by CD40-Fc treatment (Figures 4A,B). These results suggest that CD40-Fc has a greater effect on rescuing the levels of RhoA/B/C and Cdc42 *Cd40*^–/–^ cultures than rescuing PSD-95 expression. This might indicate that CD40L reverse signaling has a greater influence on controlling the expression of GTPases. Perhaps changes in the expression of these proteins might be responsible for the correct distribution of PSD-95. In this respect, it has been described that PKC isoforms acting through GEFs (Guanine nucleotide exchange factors which are activators of small GTPases) are able to modulate dendrite spine morphology (Schaffer et al., 2018). For example, PKCγ, the main PKC isoform involved in the morphological changes mediated by CD40L reverse signaling in MSNs (Carriba and Davies, 2017), phosphorylates βPIX (Pak-interacting exchange factor-β), a GEF protein that regulates Cdc42/Rac1 signaling (Shirafuji et al., 2014). Another possible explanation for the partial recovery of the expression of PSD-95 is that its expression is regulated by a combination of CD40 forward and CD40L reverse signaling. However, the available tools do not allow us to determine the level of contribution of each signaling modality in regulating PSD-95 expression, just whether it is forward or reverse signaling.

Finally, we examined whether addition of CD40-Fc to *Cd40*^–/–^ neurons cultured for 18 days was able to restore the wild type phenotype. As shown in the representative images of Figure 4C, CD40-Fc treatment, but not Fc treatment, caused a clear reduction in the number of spines. Quantification of the number and type of spines confirmed this observation (Figure 4C, bottom bar charts). The addition of CD40-Fc, but not Fc, to cultures established from *Cd40*^–/–^ mice produced a statistically significant reduction in the number of branched and mushroom spines, and an increase in the number of stubby spines. In accordance with our previous observations, there were no changes in the spine density or in the number of thin or filopodia spines. Furthermore, the reduced clustering of PSD-95 in spines in CD40-Fc-treated *Cd40*^–/–^ cultures resembled the appearance of PSD-95 observed in *Cd40*^+/+^ cultures. These results, together with our demonstration that CD40-Fc can partially restore the changes in RhoA/B/C, Cdc42 and PSD-95 expression seen in *Cd40*^–/–^ cultures, suggests that CD40L reverse signaling mediates these changes.

Overall, our findings suggest that CD40L reverse signaling participates in striatal MSN dendrite spine maturation by regulating the expression of several proteins that have been shown to control spine morphology. Our demonstration that CD40L reverse signaling influences spine maturation is not only relevant for development and functional maturation but may have implications for neurological disorders. The time course of spine maturation has a major bearing on the establishment and modification of neural circuits. Premature spine stabilization impairs synaptic plasticity. Learning spines are overproduced during early life and in adolescence, most of which are subsequently pruned together with progressive stabilization of memory spines and plastic spines. Premature maturation of dendritic spines has been implicated in the etiology of autism spectrum disorder associated with mutations in the *SYNGAP1* gene (Clement et al., 2012). Furthermore, alterations in appropriate pruning has been described in schizophrenia (Glausier and Lewis, 2013), and higher dendritic spine density has been described in depression and drug abuse (LaPlant et al., 2010). How CD40L reverse signaling influences the levels of these proteins will require further work, since regulation by a transcriptional mechanism does not seem to be involved. Moreover, it will be informative to ascertain the molecular mechanism downstream of activated CD40L that leads to changes in the expression of specific proteins, and to what extent this signaling pathways occurs locally in spines. Does the activation of CD40L by CD40 occur by an autocrine mechanism, as has been shown in low-density sympathetic neurons cultures (McWilliams et al., 2015), or does it occur by a paracrine mechanism? Another important question is whether CD40 is clipped off the membrane and is diffusible or is it membrane bound. A further interesting question is whether the influence of CD40/CD40L signaling on spine maturation is restricted to development or does it play an ongoing role in spine plasticity throughout life? Importantly, does CD40/CD40L signaling play a role in the etiology and/or progression of neurological diseases, especially given the participation of CD40/CD40L signaling in autoimmune neuroinflammatory disorders.

## Data Availability Statement

The datasets generated for this study are available on request to the corresponding author.

## Ethics Statement

The animal study was reviewed and approved by Cardiff University Ethical Review Board.

## Author Contributions

PC carried out the experimental work. SW carried out the qPCR experiments. PC and AD wrote the manuscript.

## Conflict of Interest

The authors declare that the research was conducted in the absence of any commercial or financial relationships that could be construed as a potential conflict of interest.
