# Supplementary material for: CD40L Reverse Signaling Influences Dendrite Spine Morphology and Expression of PSD-95 and Rho Small GTPases
Source: Front Cell Dev Biol. 2020 Apr 28;8:254. doi: 10.3389/fcell.2020.00254 (PMC7198883; doi:10.3389/fcell.2020.00254)
Supplement: Supplementary file 1 [file Data_Sheet_1.pdf]

CD40L reverse signaling influences dendrite spine morphology and expression of PSD-95 and Rho small GTPases

Paulina Carriba[1]*, Sean Wyatt[1] and Alun M Davies[1]

1. Neuron development, Neurosciences Department, School of Biosciences, Cardiff University, Cardiff, Wales, United kingdom.

***Correspondence**:
Paulina Carriba
paulina.carriba@gmail.com
ORCID: 0000-0002-6980-2277

Running title: CD40 and dendritic spine morphology

Key words: CD40L reverse signaling, dendritic spines, PSD-95, Rho small GTPases, Rho, Cdc42

Supplementarymaterial

Supplementary materials and methods

Quantitative PCR (qPCR)

The levels of *Psd95*, *Rac1*, *Rhoa*, *Cdc42* and *Syp* mRNAs were quantified by real-time PCR relative to a geometric mean of mRNAs for the house keeping enzymes glyceraldehyde phosphate dehydrogenase (*Gapdh*), succinate dehydrogenase (*Sdha*) and hypoxanthine phosphoribosyltransferase-1 (*Hprt1*). Total RNA was extracted from dissected striatum or MSN cultures using the RNeasy lipid mini extraction kit (Qiagen, Crawely, UK) and 5 µl was reverse transcribed for 1 h at 45°C using the AffinityScript kit (Agilent, Berkshire, UK) in a 25 µl reaction according to the manufacturer's instructions. 2 µl of cDNA was amplified in a 20 µl reaction volume using Brilliant III ultrafast qPCR master mix reagents (Agilent). PCR products were detected using dual-labeled (FAM/BHQ1) hybridization probes specific to each of the cDNAs (MWG/Eurofins, Ebersberg, Germany). The PCR primers were:
Psd95 forward: 5'-CTG TTT GAC TAC GAC AAG-3' and reverse: 5'-TCA ATT ACA TGA AGC ACA T-3'; *Rac1* forward: 5′-CCT GCT GTT GTA AAT GTC -3′ and reverse: 5′-ACT GGT TCA TTG GTT CAA-3′; *Rhoa* forward: 5′-ACA CCG ATG TTA TAT TGA TGT G-3′ and reverse: 5′-CTC GTC ATT CCG AAG GTC-3′; *Cdc42* forward: 5′-GCA AGA GGA TTA TGA CAG A-3′ and reverse: 5′-GGA CAG TGG TGA GTT ATC-3′; *Syp* forward: 5′-CCT CCT AAC TCT AGC CTT G-3′ and reverse: 5′-CCA CCC ATT TCA TCC AAG-3′; *Gapdh* forward: 5′-GAG AAA CCT GCC AAG TAT G-3′ and reverse: 5′-GGA GTT GCT GTT GAA GTC-3′; *Sdha* forward: 5′-GGA ACA CTC CAA AAA CAG-3′ and reverse: 5′-CCA CAG CAT CAA ATT CAT-3′; *Hprt1* forward: TTA AGC AGT ACA GCC CCA AAA TG and reverse: AAG TCT GGC CTG TAT CCA ACA C-3'. Dual-labeled probes were:
Psd95: 5'-FAM-CCA AGG ACT GCG GTT TCT T-BHQ1-3';
Rac1: 5'-FAM-CCT CGT TCT CGG TCC TGC CTG-BHQ1-3';
Rhoa: 5'-FAM-TCC ATT GAC AGC CCT GAT AGT TTA GAA-BHQ1-3';
Cdc42: 5'-FAM-TAC GAC CGC TAA GTT ATC CAC AGA C-BHQ1-3';
Syp: 5'-FAM- CCT CCA GCA TCG CCT GAA CTT A-BHQ1-3';
Gapdh: 5′-FAM-AGA CAA CCT GGT CCT CAG TGT-BHQ1-3';
Sdha: 5′-FAM-CCT GCG GCT TTC ACT TCT CT-BHQ1-3';
Hrpt1: 5'-FAM-TCG AGA GGT CCT TTT CAC CAG CAA G-BHQ1-3'.
Forward and reverse primers were used at a concentration of 150 nM and dual-labeled probes were used at a concentration of 300 nM. PCR was performed using the Mx3000P platform (Agilent) using the following conditions: 45 cycles of 95°C for 10 s and 60°C for 35 seconds. Standard curves were generated for each cDNA for every real time PCR run, by using serial five-fold dilutions of reverse transcribed mouse adult brain total RNA (Zyagen, San Diego, USA). Relative mRNA levels were quantified in 3 to 5 separate dissected striatum at each age and genotype and 3 separate MSN cultures at each time point and genotype. Primer and probe sequences were designed using Beacon Designer software (Premier Biosoft, Palo Alto, USA).

Supplementary figure legends

Suppl Figure 1. Expression of Rac1/2/3, Cdc42 and RhoA/B/C in *Cd40*$^{-/-}$ and *Cd40*$^{+/+}$ P10 striatal mice and in MSNs cultured 18 days. (**A**) Double labeling of DARPP-32 (green) and Rac1/2/3, Cdc42 or RhoA/B/C (all in red) and merge (yellow) in striatum of P10 *Cd40*$^{-/-}$ and

Cd40$^{+/+}$. Upper microphotographs show merge images at low magnification. Scale bars, 50 μm and 20 μm. (**B**) Expression of Rac1/2/3, Cdc42 and RhoA/B/C (all in red) in neurons cultured 18 days from E14 embryos *Cd40*$^{-/-}$ and *Cd40*$^{+/+}$. DARPP-32 (green) was used to identify MSNs in the culture. Upper microphotographs show merge images (yellow) at low magnification. Scale bars, 20 μm and 5 μm.

Suppl Figure 2. Quantitative PCR (qPCR). (**A**) Relative expression (determined by qPCR) of PSD-95, Rac, Rho and Cdc42 mRNAs at different time points in neuron cultures established from *Cd40*$^{+/+}$ and *Cd40*$^{-/-}$ mice. Graphs show mRNA levels expressed in relative units from three independent cultures at each time point and genotype normalized to three reference genes (*gapdh*, *sdha* and *hprt1*). (**B**) Relative expression (determined by qPCR) of PSD-95 and synaptophysin mRNAs in striatal tissue from animals *Cd40*$^{+/+}$ and *Cd40*$^{-/-}$ at day of birth (P0) and postnatal 5 (P5), 10 (P10) and 15 (P15). Graphs show mRNA levels expressed in relative units from three to five animals at each age and genotype normalized to three reference genes (*gapdh*, *sdha* and *hprt1*).
